# Validity of a Surveillance System for Childhood Injuries in a Rural Block of Tamilnadu

**DOI:** 10.4103/0970-0218.44650

**Published:** 2009-01

**Authors:** M Sivamani, V Balraj, JP Muliyil

**Affiliations:** Department of Community Medicine, Rajah Muthiah Medical College, Annamalai University, Chidhambaram, Tamil Nadu, India; 1 Department of Community Medicine, Christian Medical College, Vellore, India

**Keywords:** Childhood injuries, epidemiology, surveillance

## Abstract

**Background:**

Childhood injuries are increasingly getting the attention of public health experts following WHO's report on global burden of diseases. Surveillance is an important component of control of any disease and effectiveness of the surveillance system depends upon completeness of the information about occurrence of the health related events to the public health authorities.

**Aims:**

This study aimed to set up a surveillance system for childhood injuries and validate it by a survey and thereafter estimate the incidence of childhood injuries using capture recapture method.

**Settings and Design:**

Observational study design.

**Materials and Methods:**

Passive surveillance system for childhood injuries was created for 26,811 children of less than fourteen years of Kaniyambadi block and it was validated by cross sectional study at the end of surveillance period. Using these two independent information systems, capture recapture method was applied to find out the possible incidence of injuries in the given population at a given period of time.

**Statistics:**

Chi square, Lincoln Peterson formula for capture re-capture method.

**Results:**

Surveillance and survey for childhood injuries identified 13.59/1000 child-years (CI: 11.86 - 15.32) and 341.89/1000 child-years (CI: 254.46-429.33) of injury rates, respectively.

**Conclusion:**

Passive surveillance system underreports childhood injuries markedly but it does identify childhood injuries of serious nature.

## Introduction

Injury is one of the leading cause of death and burden of disease in all age groups, all over the world. Every day around the world, almost 16,000 people die from injuries. It is estimated that 5.8 million people worldwide died from injuries in 1998 which corresponds to a rate of 97.9 per 100,000 population.(1) Injuries cause over 14% of all disability adjusted life years (DALY) losses for the world's entire population, and is expected to increase as a health problem globally.(2) It is an emerging problem in developing countries.(3) However, from the beginning of mankind, injuries (un-intentional) were thought to be due to accidental ones and not preventable. William Haddon Junior challenged this view and described the ways of prevention of injuries.(4) This paves way to research in injuries.

Both developmental and behavioral characteristics of children may increase their likelihood of injury. These characteristics may be associated with an increased risk of exposure to hazards or with a decreased ability to cope with hazardous situations.(5)

Development of effective injury prevention measures depends on reliable detailed information on the incidents and outcomes of specific mechanisms of injury. In high-income countries, such data usually comes from vital statistics registries and from health care records. In low-income countries like India, such sources of data are of limited value. Many deaths are never reported to governments and information on the cause of death is limited and/or unreliable. Moreover, many ill or injured persons never receive formal medical care, making health care records an incomplete source of information.

There is, therefore, a need to study childhood injuries at community level and to develop a surveillance system for childhood injuries.(6) Hence, this study aimed to set up a surveillance system for childhood injuries and validate it by a survey and thereafter estimate the incidence of childhood injuries using capture recapture method.(7)

## Materials and Methods

### Study design

Observational study design

### Study setting

The study area for the surveillance system was Kaniyambadi Block, a rural development block in southern India with a population of just over 100,000 residents in 82 villages in an area of 182 sq kms.

### Health care delivery system

Health care in the block is provided by the Community Health Department (CHAD) through health workers at different levels. At the periphery are the Part Time Community Health Worker (PTCHW), one for every 1000 to 1500 population and Traditional Healer (TH), supervised by a Health Aide (HA), one for every 5000 population, and in turn, supervised by a Community Health Nurse (CHN) for every 15,000 population. Two doctors visit every village once a month, the nurse visits the village once in two weeks, the HA visits every village once a week. Reporting of morbidity and mortality is done by HAs, who collect information through the PTCHWs and THs, record it in appropriate registers and pass it on to the Public Health Nurse (PHN), who in turn passes it on to the statistician at the community health department for compilation. At the periphery, an Extension Worker (EW) supports the work of the PTCHW and HAs by helping to run the peripheral clinics. The primary role of this worker is developmental work in the villages.

### Study period

The first phase of the study was the development of a surveillance system. This was undertaken from January to September 2001. The second phase was a cross sectional survey to validate the surveillance system and was done from August 15^th^ to September 15^th^ of 2001.

### Study participants

For the purpose of the survey and surveillance, the case definition for childhood injuries included one or more of the following: Injuries (such as injury due to vehicle, drowning, burns, falls, bites or cut injuries.), poisoning (by substances like kerosene, medicines and insecticides.), child abuse (physical, psychological and sexual abuse.) and others (due to foreign body in the nose). Children between 0 and 14 years, permanent resident of Kaniyambadi block were eligible to participate in the study. The injuries treated by a health professional or injuries which had disabled the children for more than one day were considered as injuries for study purpose. Birth injures and iatrogenic injuries were excluded.

### Surveillance system

EWs, HAs, PTCHWs and THs in the study area were trained in the identification and reporting of childhood injuries. Training the staff of the four primary health centers in Kaniyambadi block followed this.

The case definition of childhood injuries was discussed in detail and copies of the case definition were distributed to the participants to ensure uniform reporting. Printed self-addressed forms were provided to HAs to be filled and returned while reporting childhood injury in accordance with the case definition given. Prepaid, printed and self- addressed inland letters were provided to the PHC staff to report childhood injuries. Completed forms were collected every week, entered in the injury register and were given to respective area EWs who visited the victim's house and interviewed the care taker of the injured child to gather details of the injury. Whenever there was a discrepancy between the two reports (HA and EW), the area PHN was asked to visit the house and verify. All injuries reported from January 15, 2001 to September 15, 2001 were included for analysis. At the end of the surveillance period, a cross sectional survey was done to validate the surveillance system.

### Sample size for survey

Injuries follow Poisson distribution. In Poisson distribution, we consider events and not the persons. Here, mean number of events that had occurred is considered as variance and square root of the variance is taken as standard error.(8) A pilot study was done in a near by slum. Among 125 children seen, seven (5.6%) had injuries as per the case definition. To get a precision of 20%, 100 injuries or more would be needed. Injuries less than 100, would not give 20% precision. If 5.6 injuries were found in 100 children, then to get 100 injuries 1785 children had to be seen.

### Sampling method

For the cross sectional study, eight clusters (villages) among the 82 villages of Kaniyambadi block were chosen by single stage cluster sampling method. The study villages were chosen using the 30-cluster sampling method. Since the expected variation between the clusters was considered negligible, eight clusters were selected instead of the conventional 30. This was done after identifying the first cluster from a random number. Thereafter, seven clusters (villages) were systematically identified by adding the class interval to the cumulative population of the last selected cluster. It was planned to enumerate at least 225 children in each cluster. If that number was not reached in that village, the survey continued on to the next closest village. Within each cluster, the selection of the first house for the survey was by a random method and thereafter, the next closest house was selected.

Information was sought from the caretaker of the child (mother or closest relative) regarding injury that had occurred in the previous month. For those who had suffered an injury, a four-page questionnaire was administered verbally in Tamil. Vacant houses were revisited once.

### Method of validation

Reports from the surveillance system were compared with the results from cross sectional survey. Since the two were independent sources of information, using capture-recapture method, injuries that could have been missed by both systems were calculated.

## Results

### Injury surveillance system

Over an eight-month period, January 15^th^ to September 15^th^ of 2001, 322 injuries were reported in the Kaniyambadi block, among 26,811 children (N) in the age group of 0 to 14 years. Using the inclusion and exclusion criteria, 79 cases were excluded.

Tables 1 and 2 show grouped age and sex distribution of children and injuries reported among them in the surveillance. Injury rate among 0-14 years was 13.59/1000 child- years (CI: 11.86 - 15.32).

**Table 1:** Grouped age distribution of children and their injuries included in the surveillance

Age group in years	Number of children	Number of injuries over 8 months	Child years of observation (8 months)	Injury rate/1000 child years
0-4	7996	88	63968	16.50
5-9	8967	98	71736	16.39
10-14	9848	57	78784	8.68
Total	26811	243	214488	13.59

**Table 2:** Sex distribution of children and their injuries included in the surveillance

Sex	Number of children	Number of injuries over 8 months	Child years of observation (8 months)	Injury/1000 child years
Male	13695	152	109560	16.64
Female	13116	91	104928	10.40
Total	26811	243	214488	13.59

Difference between males and females χ^2^ = 12.8, df:1; *P*=0.0003

The measurement refers to the incidence in an open cohort of children. To measure the incidence density N * 8/12 has been taken as the denominator. Even if we consider the average time contributed by the staggered entry of the newborn and staggered exit of the older children the incidence may not be beyond the confidence interval.

### Outcome of injuries

One third of injuries reported in surveillance system did not cause any disability. About 6.3, 38.3, 18.6 and 2.5% of the total injuries resulted in disability for 1 day, 2-7 days, 8-30 days and more than 30 days, respectively. Injuries led to death were 2.8%. The injury specific mortality rate was 39.16/100,000 child-years (CI: 10.07-68.25).

Fewer injuries were reported among girls compared to boys.

## Cross Sectional Survey

### Clusters

There were 2141 children under 14 years in the eight village clusters. The clusters were of comparable sizes. According to 2001 census, the population surveyed has covered 80% of population of each village except one where it covered only 37%.

Among the 2141 children, 61 injuries were identified in the survey as per the case definition. This works out to a morbidity rate of 341.89/1000 child years. (CI: 254.46-429.33). Tables 3 and 4 show grouped age and sex distribution respectively of children and their injuries identified in the survey.

**Table 3:** Grouped age distribution of children and their injuries included in the survey

Age group in years	Number of children	Number of injuries over one month	Injury rate/1000 child years
0-4	653	16	294.02
5-9	691	19	329.95
10-14	797	26	391.46
Total	2141	61	341.89

**Table 4:** Sex distribution of children and their injuries included in the survey

Sex	Number of children	Number of injuries in one month	Injury rate/1000 child yrs
Male	1097	39	426.61
Female	1044	22	252.87
Total	2141	61	341.89

### Outcome

There were no deaths. Half of the injuries did not cause disability. Nearly 14.8%, 27.8% and 3.3% of the total injuries resulted in disability for 1 day, 2-7 days and 8-30 days, respectively.

### Validation

Primary goal of the surveillance system is to collect representative data on the occurrence of the disease in a population that enables public health officials to make decisions on what interventions are needed to meet their objectives to control the disease. Effectiveness of any health information system depends on completeness of the data.(9) To determine the completeness of the coverage of the surveillance and survey, the Lincoln Peterson capture - recapture method was used to calculate the probable number of injuries in the given population.(10) Data from the two systems were matched using unique ID number of the children. Assuming that the surveillance and survey were independent, the number of injuries identified by the both the systems, and those missed by both the systems were analyzed. Using Lincoln Peterson's formula:

$$\begin{array}{c} \mathrm{Probable number of injuries in the population (N)}\\ =\frac{\mathrm{Injuries identified by system 1 alone}\times \mathrm{Injuries identified by system 2 alone}}{\mathrm{Injuries identified by both}} \end{array}$$

N = 3 × 61/2 = 91

Table 5 shows that hypothetically 91 injuries could have occurred in the survey area, only two injuries were picked up by both the systems and both had missed 29 injuries.

**Table 5:** Capture recapture method

		Surveillance		
			
		(+)	(-)	
Survey	(+)	2	59	61
	(-)	1	29	30
Total		3	88	91

(+) Injuries identifies by the system, (-) Injuries not identified by the system

## Discussion

Cross sectional survey showed that the injury rate in age group 0 to 14 years was 341.89/1000 child-years. Surveillance in Kaniyambadi on the other hand showed an injury rate among 0-14 years of 13.59/1000 child-years, a marked under reporting through a passive system that had missed minor injuries

The theoretical possibility using the capture recapture method, showed there could have been as many as 91 injuries in the study population, but only three injuries were identified by the surveillance system and 61 injuries were identified by the survey from the same population in the same period. Among the 61 injuries, only 2 injuries were serious enough to disable the child for more than a week and they were identified by the surveillance system. This shows that only serious injuries were reported to a passive surveillance system.

Twenty percent of the reported cases in the cross sectional survey was later interviewed to determine why the injuries had not been identified by the surveillance system. The reasons stated by some of the respondents were that, “health personnel (HA) from CHAD visited the area only once in 15 days and minor injuries heal before that.” “Health aides did not stay in the village and hence they could not be approached immediately for treatment. Emergency medical services were not provided by CHAD and hence there was no point in reporting injury unless it was specifically asked for and private practitioners were available to fill this role”.

## Conclusion

Cross sectional survey in rural Tamilnadu shows that injury rate in 0-14 years was 341.89/1000 child-years. Passive surveillance in the same area in the same age group showed an injury rate of 13.59/1000 child-years and a mortality rate of 39.16/100,000 child-years due to injuries. Passive surveillance for childhood injuries does provide information about the more serious injuries but trivial injuries do not get reported.
